# Identifying Glioblastoma Gene Networks Based on Hypergeometric Test Analysis

**DOI:** 10.1371/journal.pone.0115842

**Published:** 2014-12-31

**Authors:** Vasileios Stathias, Chiara Pastori, Tess Z. Griffin, Ricardo Komotar, Jennifer Clarke, Ming Zhang, Nagi G. Ayad

**Affiliations:** 1 Department of Human Genetics & Genomics, University of Miami Miller School of Medicine, Miami, Florida, 33136, United States of America; 2 Department of Psychiatry and Behavioral Sciences, Center for Therapeutic Innovation, University of Miami Miller School of Medicine, Miami, Florida, 33136, United States of America; 3 Department of Epidemiology and Biostatistics, and Institute of Bioinformatics, University of Georgia, Athens, Georgia, 30602, United States of America; 4 Department of Neurosurgery, University of Miami Miller School of Medicine, Miami, Florida, 33136, United States of America; 5 Department of Food Science and Technology, Department of Statistics, University of Nebraska, Lincoln, Nebraska, 68588, United States of America; Harbin Medical University, China

## Abstract

Patient specific therapy is emerging as an important possibility for many cancer patients. However, to identify such therapies it is essential to determine the genomic and transcriptional alterations present in one tumor relative to control samples. This presents a challenge since use of a single sample precludes many standard statistical analysis techniques. We reasoned that one means of addressing this issue is by comparing transcriptional changes in one tumor with those observed in a large cohort of patients analyzed by The Cancer Genome Atlas (TCGA). To test this directly, we devised a bioinformatics pipeline to identify differentially expressed genes in tumors resected from patients suffering from the most common malignant adult brain tumor, glioblastoma (GBM). We performed RNA sequencing on tumors from individual GBM patients and filtered the results through the TCGA database in order to identify possible gene networks that are overrepresented in GBM samples relative to controls. Importantly, we demonstrate that hypergeometric-based analysis of gene pairs identifies gene networks that validate experimentally. These studies identify a putative workflow for uncovering differentially expressed patient specific genes and gene networks for GBM and other cancers.

## Introduction

Glioblastoma multiforme (GBM) is the most common malignant adult brain tumor, comprising 15.6% of all central nervous system tumors [1]. The median two-year survival is 13.7%, and disease remission following standard therapy occurs within 6.9 months. [1], [2] Treatment includes surgical resection followed by radiation and temozolomide (TMZ) administration. However, TMZ resistance is nearly universal, suggesting that we need to understand the genetic landscape of GBM tumors more extensively in order to uncover more effective therapies [3].

Recent developments in oncogenomics point to a highly heterogeneous genomic landscape in GBM [4], [5]. Importantly, this heterogeneity necessitates genome and transcriptome analyses of each tumor individually in the hopes of discovering patient specific therapies [6]. However, discovering patient–specific transcriptional alterations is difficult given the low patient sample size (n = 1). This is especially true when using RNA sequencing given the discordance of different RNA-Seq alignment and analysis algorithms when sample size is small [7].

One possibility to increase the available sample size is to utilize transcriptome data in publicly available databases as a reference. For instance, The Cancer Genome Atlas (TCGA) has performed gene expression microarray analysis in over 400 GBM patients examining them using two different platforms (Agilent and Affymetrix). Thus, it is possible to use these data as a reference set, to compare the RNA sequencing results from a single tumor sample and identify differentially expressed genes and gene networks. Utilizing a novel bioinformatics pipeline we were able to perform a patient-specific analysis of the GBM transcriptome based on the overlap between our RNA-Seq data and the TCGA GBM data. This approach allowed us to identify and filter out potential artifacts due to low sample size.

In this report we identified a patient specific list of differentially expressed genes (DEGs), which can be used as input for multiple types of analyses including gene co-expression networking. Genes that co-express across multiple samples are often implicated in similar functions [8] and many disease-associated genes have been discovered through co-expression network analysis [9]. Most methods used in previous studies are based on the calculation of correlation coefficients (usually Pearson) of gene pairs as an indication of co-expression. Furthermore, either weighted [10] or unweighted [11] processes involving the proposed connections between genes are used to determine the significance thresholds for assigning a connection between any two nodes (i.e., genes) in the resulting network. Our studies suggest that utilizing correlation and hypergeometric tests identifies experimentally validated gene connections, which can potentially assist in discovering patient specific therapies.

## Materials and Methods

### RNAseq quality control and genome mapping

We performed whole transcriptome sequencing on two GBM tumors (GBM17 and GBM31) and two control samples from epileptic patients using the Illumina HiSeq sequencing platform. Preliminary screening was performed in FastQC (FASTQC 2012) and BLAST [12] to assess the sequence read quality and to filter for potential adapter contamination. Low quality reads were trimmed and adapters were removed in downstream analysis. Remaining reads from each sample were mapped to the human genome using TopHat Version 2.0.4 [13], [14].

After read trimming, samples GBM17, GBM31, Control16, and Control34 had 87.18%, 78.56%, 86.79%, and 92.31%, reads mapped, respectively. For each sample, we also assessed the distribution of genes that mapped to the human genome in order to gauge the quality of the experiment. GBM17, GBM31, and Control 34 yielded approximately 15,000 genes with nearly 100% transcript coverage in the reference human genome. Control16 had only 8,085 mapped genes. Only the common 8,085 genes were used in the 4 differential expression analyses that followed.

### RNAseq Differential Expression Evaluation

Four differential expression analysis tools, rather than one single tool, were applied to the data and the results from all four tools were compared. This yielded results that are relatively robust to both varying tool approaches to sequencing depth normalization and statistical tests employed, as well as to inherent variability in the RNA-Seq data. The four methods used were: baySeq 1.10.0 [15], DESeq 1.8.3 [16], edgeR 2.6.12 [17], and Cuffdiff 2 [13].

### TCGA Microarray Expression Data

Two expression datasets were collected from The Cancer Genome Atlas (TCGA) in 07/02/2014 (https://tcga-data.nci.nih.gov/tcga/). The first dataset contained tumor specific expression data from 433 glioblastoma patients (P1-P433) and the second dataset contained brain tissue expression data from 10 epileptic patients. All samples were analyzed with both the AgilentG4502A Microarray Platform and the Affymetrix HG-U133 Microarray Platform. The Level 3 (processed) data for these samples were downloaded and further analyzed. The data processing and quality control were performed by The Broad Institute's TCGA workgroup. The AgilentG4502A Level 3 data consisted of the lowess normalized log2 expression values [18]. The Affymetrix HG-U133 Data were RMA normalized and hence are on a log2 scale [19].

Differentially expressed (DE) genes between glioblastoma patients and epileptic controls were identified by using the *limma* package in R [20] (moderated t-statistic and also the Benjamini and Hochberg's method to control for FDR). Out of the 17,814 genes detected by the AgilentG4502A Platform, 6,889 genes were found to be differentially expressed (FDR adjusted p-value<0.05). Out of the 12,042 genes detected by the Affymetrix HG U133 Platform, 7,503 genes were differentially expressed (FDR adjusted p-value<0.05). Filtering for genes that had a minimum fold change was not performed at this step, as it would only depict the gene expression tendency according to the average of all the patients and may conceal any gene expression patterns that characterize particular subgroups of patients. Minimum fold change filtering was performed at a later stage of the analysis. Patient specific gene expression fold change was calculated for each patient relative to the average gene expression in the TCGA tissue specific controls.

### Hypergeometric Test

A hypergeometric-based test was used to assess the significance of co-expression across samples between two genes of a gene pair. The rationale behind using the hypergeometric test was that if 2 genes had any biological and/or functional association, they would be found co-expressed in a higher number of samples than expected by chance. We tested under the null hypothesis that the property of a gene to be DE in one sample is independent of the property of another gene to be DE in the same sample. The p-value was derived from the hypergeometric function below where f: total number of patients, d: number of patients for which Gene1 is DE, g: number of patients for which Gene2 is DE, n: number of patients for which both Gene1 and Gene2 are DE 
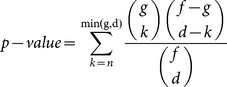
Our pipeline and the scripts for calculating the Pearson Correlation Coefficient and the Hypergeometric test can be found in (S4 Fig., S1 File).

### Creating Networks

Networks from the selected gene pairs were created using the open source Cytoscape 3.1.0 as described in [21]. Further, the STRING9.1 Protein-Protein Interaction Platform [22] was employed to verify the functional relevance of the discovered networks.

## Results

### Identifying genes differentially expressed in GBM via RNA sequencing and TCGA enrichment

**Figure 1:**
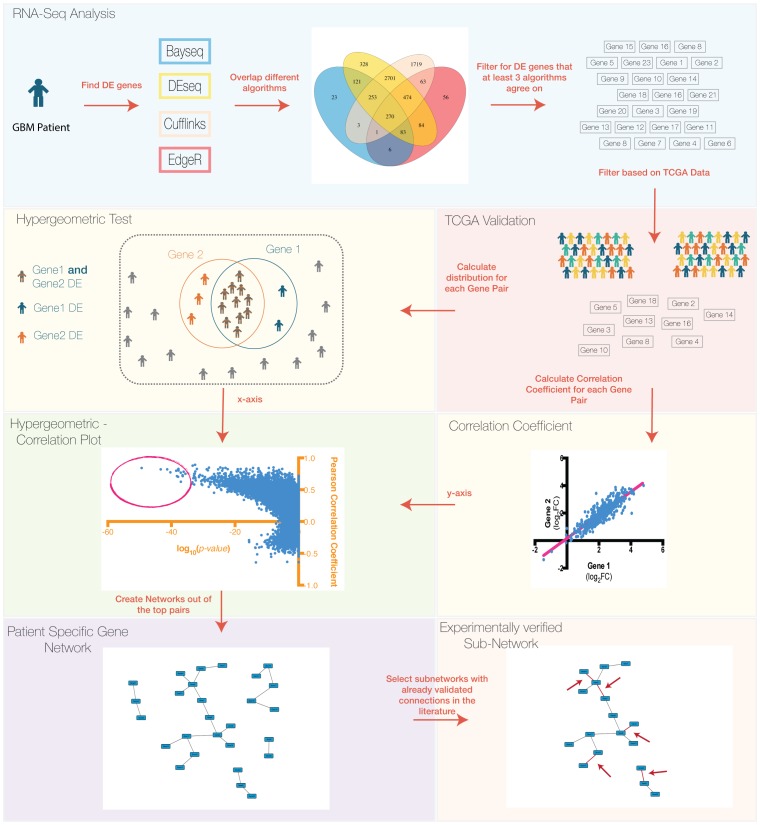
Pipeline for identifying patient-specific gene association in GBM. Our first step in our pipeline is to identify Differentially Expressed (DE) genes that are represented in 3 out of 4 algorithms. Next, we filter this DE gene list for those genes that overlapped with DE genes in the TCGA GBM Database. We then calculate the Correlation Coefficient and a hypergeometric p-value for every gene pair. Finally, by selecting the gene pairs with the highest correlation values we create a patient specific gene correlation network, which can be experimentally verified. As a starting point for our experiments, we can use the sub-networks in which, already verified connections exist in the literature.

We performed RNA sequencing on 2 GBM tumors (GBM17 and GBM31) and 2 epilepsy control tissues (Control16 and Control34) using the Illumina HiSeq platform. Focusing on patient specific transcriptionally expressed genes, we compared the transcriptome of each tumor individually with the two controls. In order to ameliorate the increased uncertainty of the different algorithms due to low sample size [7], we used four, rather than one, differential expression tools (baySeq, Cuffdiff, EdgeR and DESeq), as described in the Materials and Methods Section. As expected, the four algorithms yielded mostly non-overlapping lists of differentially expressed genes. We defined a given gene as ‘differentially expressed (DE)’ if at least three of the four algorithms called the gene as differentially expressed. By performing this type of analysis, we identified 1,081 DE genes for GBM17 and 967 genes for GBM31, respectively (Fig. 1, S1 Fig.).

In order to assess whether these results represent the phenotype of interest (GBM) or individual sample variability, we compared our RNA sequencing results to results from a greater number of samples, namely, the TCGA microarray dataset. Previous studies have shown that RNA-Seq results can be compared to those derived from microarrays [23], [24], [25]. We created a TCGA DE gene list by comparing TCGA patients and TCGA controls. In order to avoid any platform specific biases, we selected only TCGA patients (n = 433) and controls (n = 10) that were analyzed with both microarray platforms (Agilent and Affymetrix). When we examined these data, we found that 5,200 genes were found by both platforms to be DE and concordant, i.e, *not* upregulated in one platform and downregulated in another platform. Of these 5,200 DE genes, we found that 585 are DE in GBM17 and 514 are DE in GBM31 (S1 Fig.). In S2 Fig. we can see that when using the consensus of at least 3 algorithms, the number of common DE genes between our RNAseq data and the TCGA data remains relatively the same. On the other hand, when only one algorithm was taken into account, the number of overlapping genes (between RNAseq and TCGA) was greatly dependent on the fold change threshold selection. Therefore, we opted for the 1.2 fold change threshold, in an attempt to obtain a high and significantly relevant number of genes.

### Identifying Gene Pairs and Networks Using Hypergeometric Test Analysis

As acquired drug resistance is one of the main reasons for the ineffectiveness of current glioblastoma treatment, the identification and simultaneous inhibition of multiple therapeutic targets is essential for identifying effective combination therapies. However, the large genetic variation among glioblastoma tumors necessitates a more patient-specific approach when identifying potential therapeutic targets. For this, we tried to create gene networks based only on genes that were DE in that specific RNA-Seq sample. After determining the DE genes for each sample, and ‘filtering’ or selecting those that are also DE in the TCGA data, we searched for any significant co-expression among these patient-specific DE genes in the TCGA data. Our hypothesis was that if there were any functional association between gene pairs, then this association could be observed based upon their co-expression in the TCGA population. We utilized two methods to identify co-expression. First, the Pearson Correlation Coefficient (PCC) was used to determine if the expression level of one gene could be indicative of the expression level of the second gene. Next, a hypergeometric test was used to calculate the probability of a pair of genes being both DE in a specific number of patients by chance. Gene pairs with a low p-value indicate the tendency of these genes to be DE together in a higher number of patient samples than we would expect by chance. For every gene pair, we plotted both its PCC and its hypergeometric probability in the TCGA population (S1 Fig.). Our results indicate that pairs of genes that are DE in a larger proportion of patient samples also have more highly correlated expression values across the total TCGA GBM cohort. Finally, as outliers heavily influence the PCC, we calculated the Spearman Correlation Coefficient for every gene pair as well. As shown in S3 Fig. we found that gene pairs with high PCC also have a very high Spearman Correlation Coefficient, indicating that the PCCs we calculated were generally not the byproduct of outliers.

**Figure 2:**
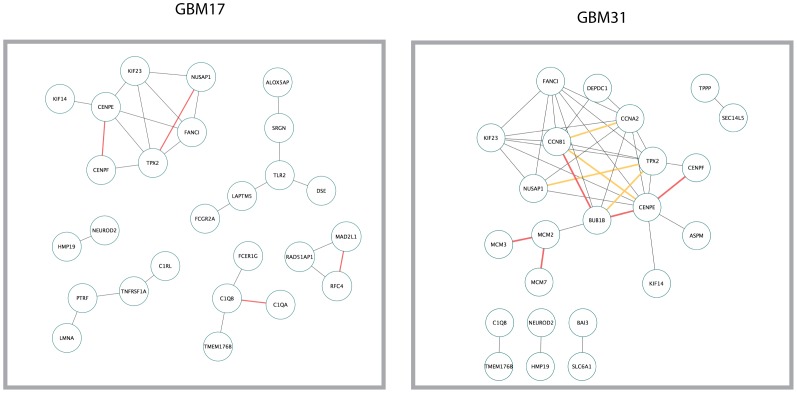
Correlation networks created by using the top gene pairs for each patient. The number of connections we identified were compared to those previously described in the literature (red). Yellow indicates connections, which were identified in protein-protein interaction databases.

**Figure 3:**
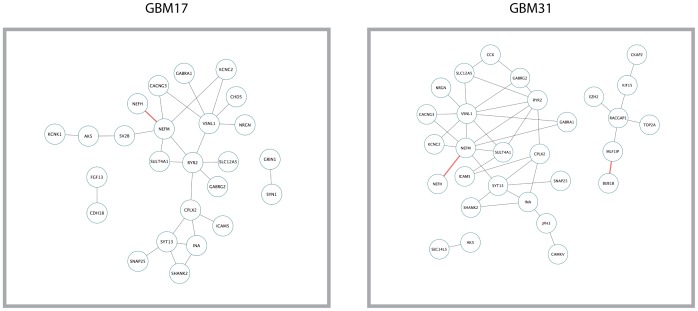
Gene Networks created by Pairs with high PCC (greater than 0.7) and high hypergeometric p-value yield less experimentally verified interactions. The number of connections identified was calculated for gene pairs with high PCC and high hypergeometric p-values. These connections were then compared to those identified in the literature. Note that few connections were found to be experimentally validated.

As our aim was to define patient-specific and glioblastoma-relevant gene networks, we used the most statistically significant gene pairs in each RNA-Seq sample in order to reconstruct the networks. Our most significant gene pairs (S1 Table) consisted of the ones that had a PCC above 0.7 and were in the lowest 40% of the range of the hypergeometric log_10_ scale ([−46.15, −27.69] for GBM17 and [−44.72, −26.83] for GBM31). These gene pairs were selected for each patient sample separately and were imported to Cytoscape [26] where the network was created. In order to verify the functional relevance of these networks, we employed the STRING9.1 Protein-Protein Interaction Platform [22]. STRING 9.1 can take into account a variety of sources in order to reconstruct networks out of a given gene list. For our study, we only chose the conservative options of searching in Databases, Experiments and Text-mining. To ensure the accuracy of the text-mining algorithm, we manually verified the text mining output. As shown in Fig. 2, there is overlap between the networks created from our pipeline and gene associations indicated by STRING 9.1. To examine whether the hypergeometric test yielded greater experimentally verified interactions, we kept the PCC filter (>0.70) constant but varied the hypergeometric test p-values. As before, we compared our gene networks with curated interaction data via STRING 9.1. As shown in Fig. 3 and S2 Table, fewer experimental data validated the gene network connections created by these gene pairs when we only used a PCC filter. Thus, our studies suggest that a hypergeometric distribution test can potentially enrich more experimentally verified gene pairs.

### Identifying Epigenetic-Signaling Pathway Interactions Based on Hypergeometric Test Analysis

**Figure 4:**
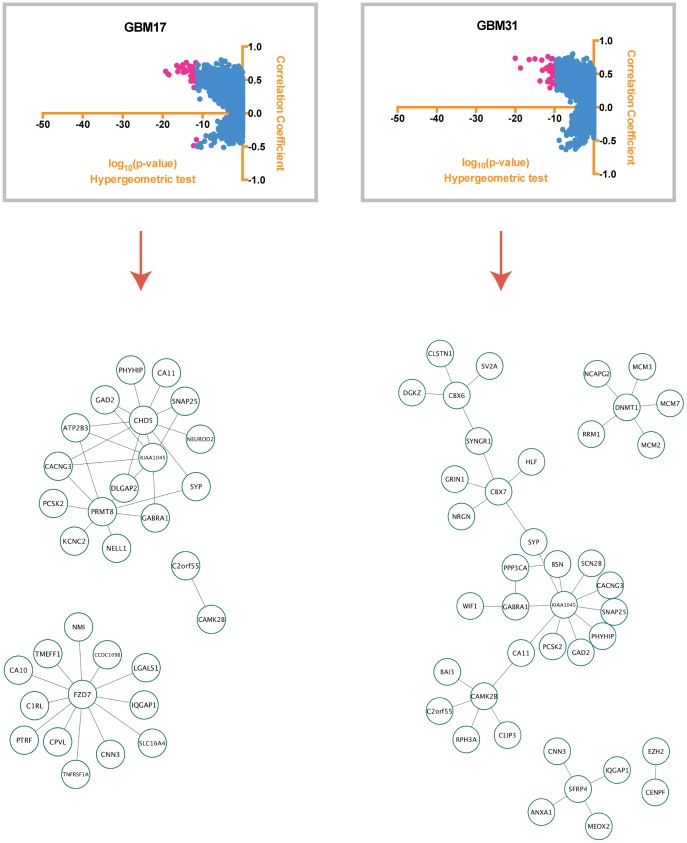
Correlation networks identified that intersect epigenetic pathways/signaling pathways with patient specific DE genes. Connections were calculated for gene-gene pairs emanating from epigenetic pathways or genes in the Notch, SHH, or WNT pathways and genes that were Differentially Expressed in each patient.

Several studies suggest that epigenetic and signaling pathways control GBM progression [27]. We therefore reasoned that the gene networks robustly uncovered by the bioinformatics pipeline described above would potentially include the interactions of DE genes involved in signaling or epigenetic pathways. To test this, we created a discrete gene list consisting of 464 genes (through KEGG Pathways) (S3 Table) that encoded for either epigenetic modulators, or genes implicated in the Notch, Shh and Wnt Pathways. Out of the 464 Epigenetic/Pathway (E/P) related genes, 418 genes were differentially expressed in the TCGA GBM data from the Agilent Microarray Platform. In order to find the non-random co-expression of E/P genes together with genes that were differentially expressed on each patient, we employed our pipeline. As shown in Fig. 4, the more statistically significant gene pairs have a higher expression correlation in the whole population of 433 GBM patients. These studies yielded potential interactions of signaling pathways or epigenetic regulators with GBM patient specific DE genes.

## Discussion

Combination therapies that effectively disrupt multiple pathways are likely to reduce GBM recurrence. However, identifying these therapies is contingent upon uncovering patient-specific genomic and transcriptomic alterations. One major issue is how to generate robust analytical results from gene expression data when sample size is low. To circumvent this problem, we elected to employ multiple different RNA-Seq algorithms and compare the results common to most algorithms with results based on the transcriptional profile of over 400 GBM tumors analyzed by TCGA. With this approach, we identified genes that not only were differentially expressed in our patient specific sample but also were differentially expressed in a significant number of TCGA patient samples. These true positive genes were subsequently used to create gene co-expression networks to identify any potential functional similarity. By using a hypergeometric test in addition to the PCC, we identified experimentally validated gene-gene interactions.

One gene network that has been experimentally verified in multiple biological contexts is the highly conserved MCM (Mini-Chromosome Maintenance) network containing the DNA replication licensing factors MCM2, MCM3, and MCM7. We identified this network as overexpressed in GBM sample 31. With the hypergeometric test, we detected a high probability of the MCM subunits to be overexpressed in the same patient samples in the TCGA dataset and were able to distinguish them from other gene pairs that only showed a high correlation coefficient (but little co-occurrence within patients). Importantly, the MCM pathway has been implicated in multiple cancers including GBM [28], [29]. Thus, our pipeline can be utilized to identify DE gene-gene interactions and networks in GBM that have a higher likelihood of validation relative to interactions and networks identified by correlation alone.

The stringency of our filtering of the DE genes from any given patient, however, is likely to also yield false negatives. For instance, we chose to only explore DE genes that were detected in at least 3 out of 4 RNA-Seq algorithms, which may have excluded some true positives. It may be informative to use other statistical methods in addition to the hypergeometric test to determine whether there are alternative approaches that would also yield results that are enriched for experimentally verified gene networks. In addition any proposed pipeline will involve subjective choices of thresholds and criteria for significance. We have made reasonable choices, but it is possible that moving to nonparametric and permutation-based results to establish a more systematic approach to threshold selection could lead to pipeline improvements. Nonetheless, our studies identify a robust method for identifying patient-specific gene networks, which can inform the design of effective combination therapies.

We implemented this method to identify potential targets for combination therapies involving epigenetic pathways and the Notch, SHH, and WNT pathways. Our recent studies and those from other laboratories suggest that epigenetic pathways contain attractive new therapeutic targets in GBM [30], [31]. Similarly, the Notch, SHH, and WNT pathways are implicated in GBM cell growth and have been linked to epigenetic pathways [27]. Thus, we reasoned that identifying interaction nodes between either signaling pathways or epigenetic modulators and DE genes could lead to potential synergy in reducing tumor growth. We identified possible nodes of interaction among epigenetic and signaling pathways using our pipeline. Interestingly, we identified possible interaction of the DNA methyltransferase DNMT1 with the MCM proteins (Fig. 4). However, these interactions have not been experimentally verified according to STRING 9.1. Nonetheless, they may exist in some GBM patients as DNMT1 has been shown to be a potential GBM therapeutic target whose inhibition leads to an increase of several tumor suppressor genes [32], [33], [34]. Future studies will experimentally verify whether drug combinations that target these gene-gene networks are useful clinically for treating GBM.

## Supporting Information

S1 Fig **Overlap of DE genes calculated by 4 RNAseq Algorithms (EdgeR, Bayseq, Cufflinks, DEseq).** We analyzed genes that were shown to be DE by at least 3 out of the 4 algorithms (* symbol). We then filtered for genes that were shown to be DE by both Microarray Platforms in the TCGA GBM cohort and also were shown to have a |fold change| >1.2. The Pearson Correlation Coefficient and the p-value of the hypergeometric test were then plotted for every gene pair.
(TIF)

S2 Fig **Fold Change dependent overlap of DE genes calculated by 4 RNAseq Algorithms and the TCGA Database.** The fold change threshold did not change the number of overlapped genes between the RNAseq and the TCGA analysis, when the consensus of 3+ RNAseq algorithms was used.
(TIFF)

S3 Fig **Comparison between two Correlation Coefficients.** The Pearson and the Spearman Correlation Coefficients were calculated for every gene pair. In both patients gene pairs with high Pearson Correlation Coefficient show also high values for the Spearman Correlation Coefficient.
(TIF)

S4 Fig **Pipeline for calculating the Hypergeometric Distribution and the Pearson Correlation Coefficient for every gene pair.**
(TIFF)

S1 Table **Gene pairs consisting of the ones that had a PCC above 0.7 and were in the lowest 40% of the range of the hypergeometric log_10_ scale.**
(XLSX)

S2 Table **Gene pairs with a PCC above 0.7 and the highest hypergeometric log_10_ scale values.**
(XLSX)

S3 Table **Gene list containing 464 genes are either epigenetic modulators, or genes implicated in the Notch, Shh and Wnt Pathways (though KEGG Pathways).**
(XLSX)

S1 File **Perl scripts used in the pipeline.**
(TXT)
