# Supplementary figures and images for: Identifying Glioblastoma Gene Networks Based on Hypergeometric Test Analysis
Source: PLoS One. 2014 Dec 31;9(12):e115842. doi: 10.1371/journal.pone.0115842 (PMC4281219; doi:10.1371/journal.pone.0115842)

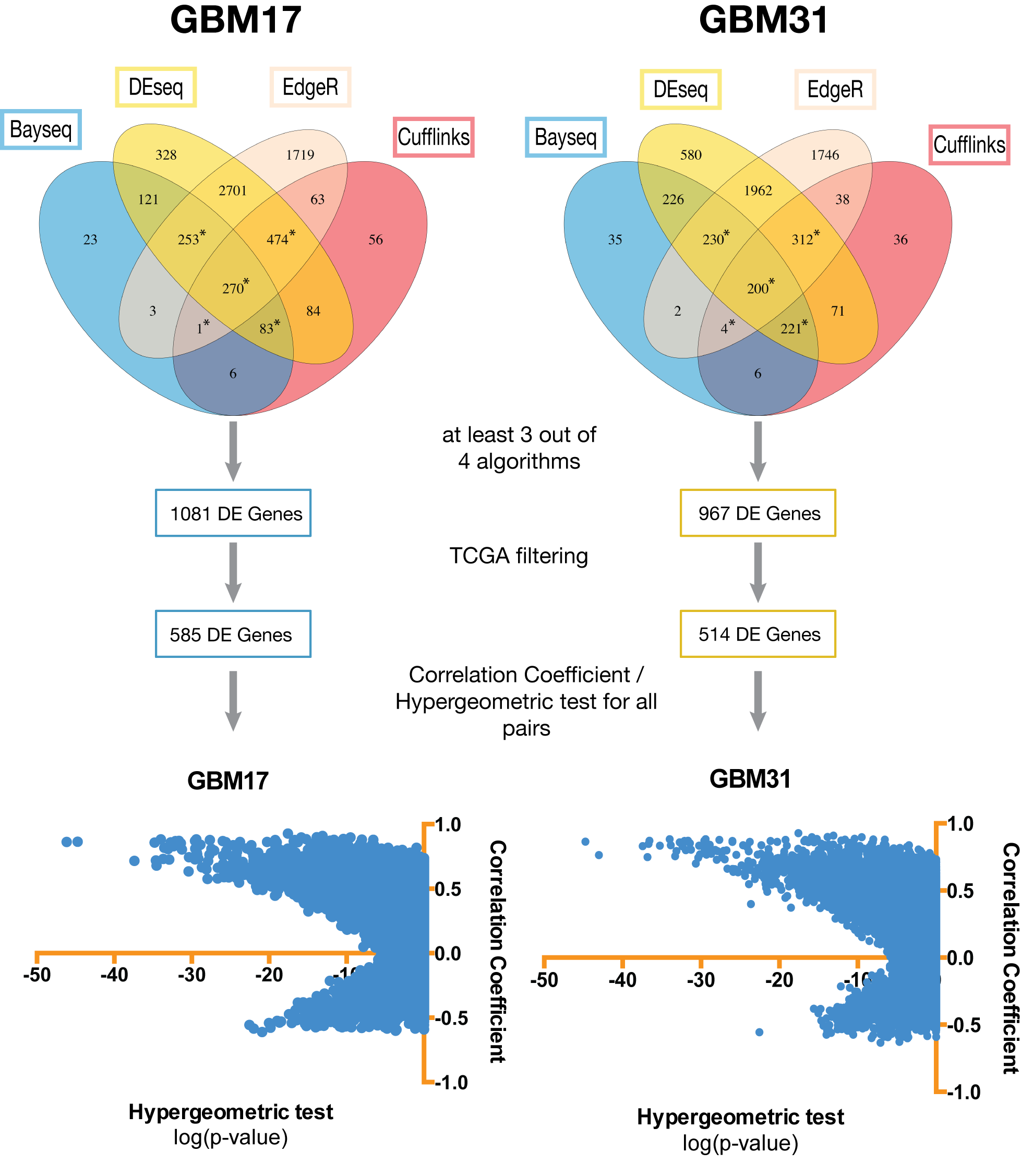

Supplement: S1 Fig — Overlap of DE genes calculated by 4 RNAseq Algorithms (EdgeR, Bayseq, Cufflinks, DEseq). We analyzed genes that were shown to be DE by at least 3 out of the 4 algorithms (* symbol). We then filtered for genes that were shown to be DE by both Microarray Platforms in the TCGA GBM cohort and also were shown to have a |fold change| >1.2. The Pearson Correlation Coefficient and the p-value of the hypergeometric test were then plotted for every gene pair. (TIF) [file pone.0115842.s001.tif]

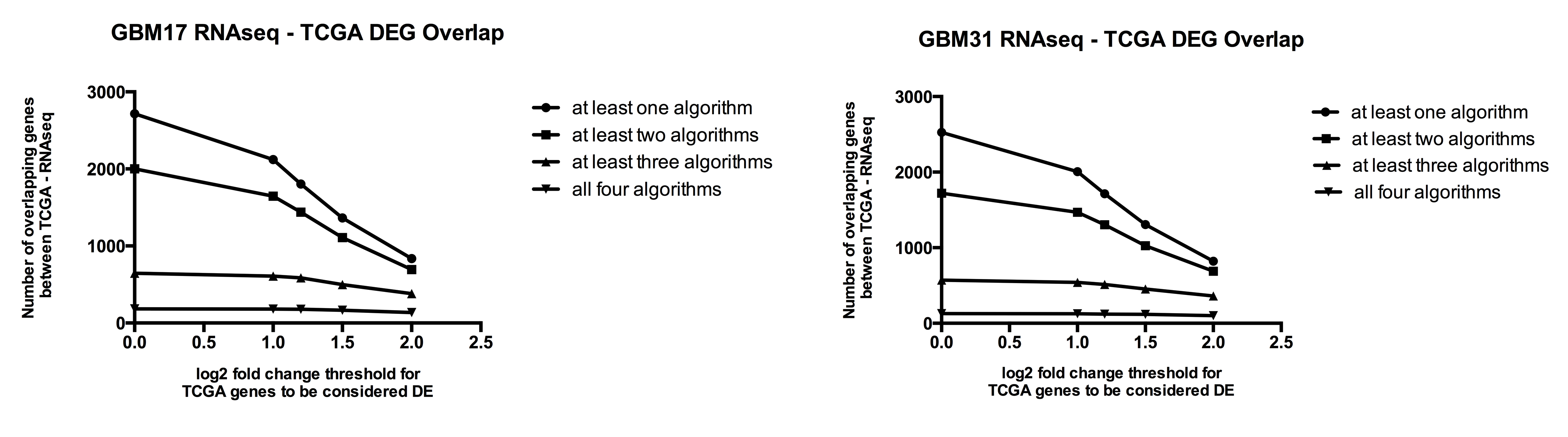

Supplement: S2 Fig — Fold Change dependent overlap of DE genes calculated by 4 RNAseq Algorithms and the TCGA Database. The fold change threshold did not change the number of overlapped genes between the RNAseq and the TCGA analysis, when the consensus of 3+ RNAseq algorithms was used. (TIFF) [file pone.0115842.s002.tiff]

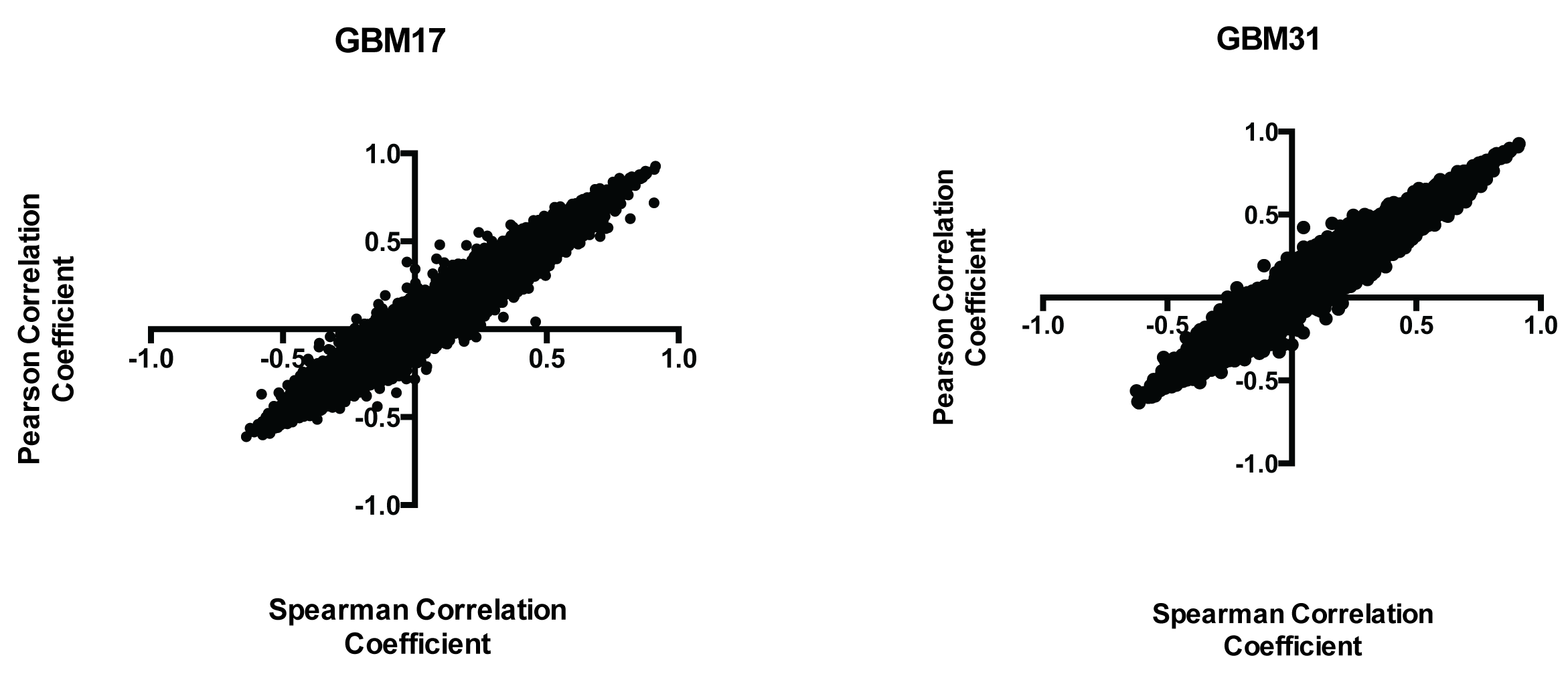

Supplement: S3 Fig — Comparison between two Correlation Coefficients. The Pearson and the Spearman Correlation Coefficients were calculated for every gene pair. In both patients gene pairs with high Pearson Correlation Coefficient show also high values for the Spearman Correlation Coefficient. (TIF) [file pone.0115842.s003.tif]

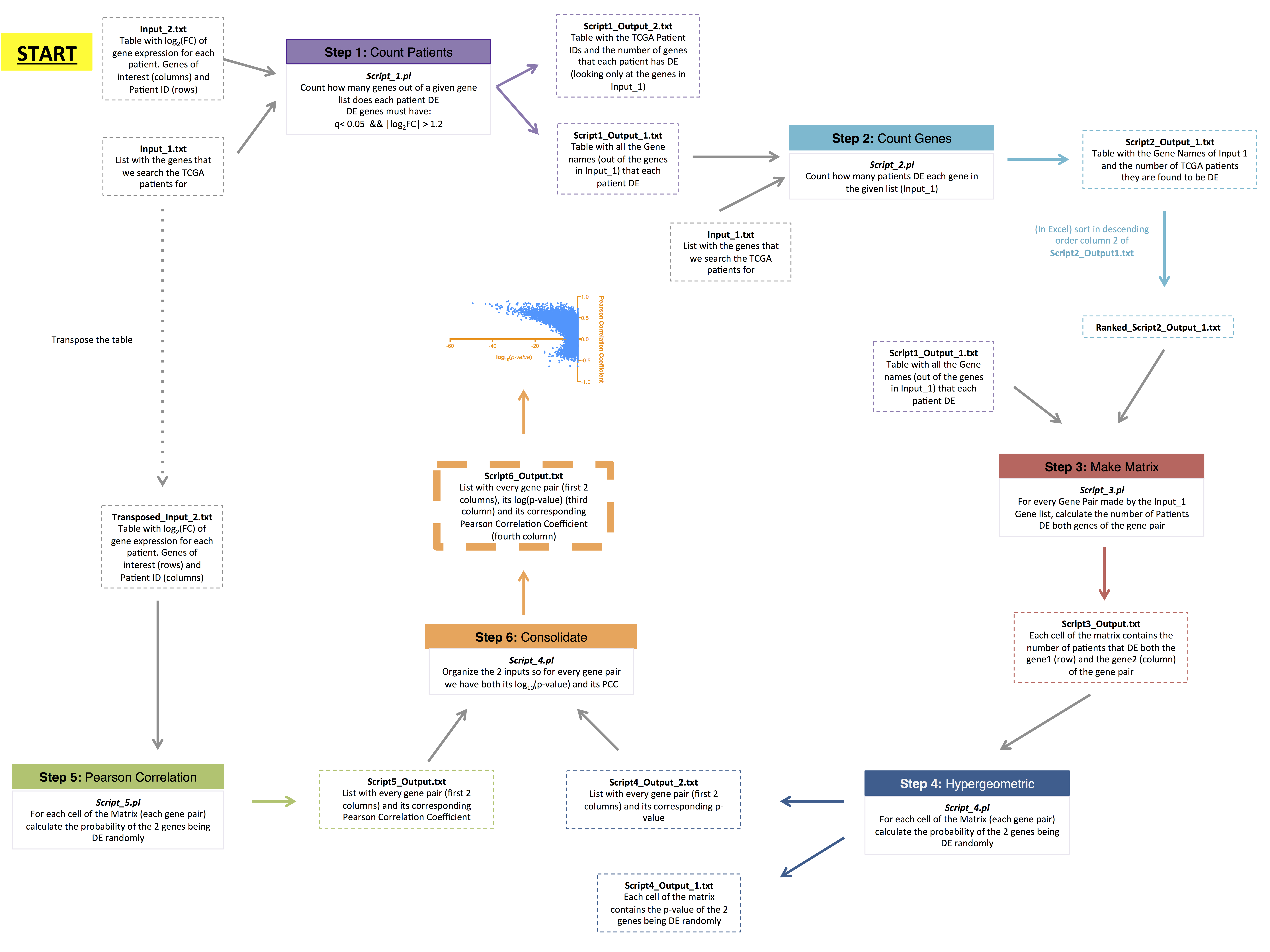

Supplement: S4 Fig — Pipeline for calculating the Hypergeometric Distribution and the Pearson Correlation Coefficient for every gene pair. (TIFF) [file pone.0115842.s004.tiff]
